# Semisolid Metal Processing Techniques for Nondendritic Feedstock Production

**DOI:** 10.1155/2013/752175

**Published:** 2013-09-08

**Authors:** M. N. Mohammed, M. Z. Omar, M. S. Salleh, K. S. Alhawari, P. Kapranos

**Affiliations:** ^1^Department of Mechanical and Materials Engineering, Faculty of Engineering and Built Environment, Universiti Kebangsaan Malaysia (UKM), 43600 Bangi, Selangor, Malaysia; ^2^Department of Materials Science and Engineering, The University of Sheffield, Sir Robert Hadfield Building, Mappin Street, Sheffield S1 3JD, UK

## Abstract

Semisolid metal (SSM) processing or thixoforming is widely known as a technology that involves the formation of metal alloys between solidus and liquidus temperatures. For the procedure to operate successfully, the microstructure of the starting material must consist of solid near-globular grains surrounded by a liquid matrix and a wide solidus-to-liquidus transition area. Currently, this process is industrially successful, generating a variety of products with high quality parts in various industrial sectors. Throughout the years since its inception, a number of technologies to produce the appropriate globular microstructure have been developed and applied worldwide. The main aim of this paper is to classify the presently available SSM technologies and present a comprehensive review of the potential mechanisms that lead to microstructural alterations during the preparation of feedstock materials for SSM processing.

## 1. Introduction

**Figure 1:**
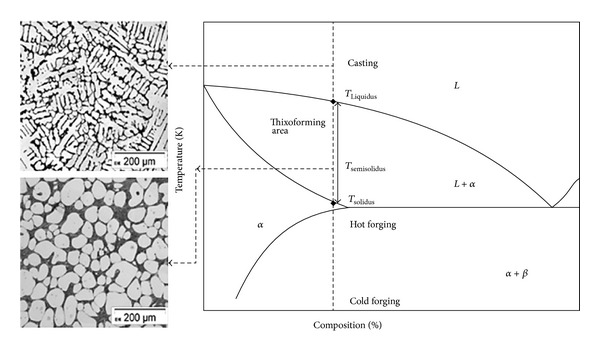
Micrograph of dendritic and globular structures in a semisolid alloy; simple eutectic phase diagram for steel grade X210CrW12 [4].

Semisolid metals processing (SSM), also known as thixoforming, is a technology for metal forming that was patented by Spencer during his Ph.D. studies under the supervision of Prof. Flemings at the Massachusetts Institute of Technology (MIT) in the early 1970s [1]. Nowadays, the thixoforming process has found manufacturing applications on a number of applications due to its ability to deliver production of high quality parts at costs that are comparable to or lower than conventional forming techniques such as casting or forging. Some examples of these applications are engine suspension mounts and steering knuckles for some automobile brands [2, 3]. During the last 40 years of research efforts, a lot of different materials have been developed and used with this process. For successful SSM processing, the microstructure should have solid near-globular grains with a wide transition area from solidus to liquidus, as shown in Figure 1 [4]. These microstructures impart thixotropic properties in slurries; that is, they have shear and time dependent flow properties.

A unique property of the flow behavior in SSM is related to the non-Newtonian behavior of an alloy where, in this case, when the material state is 50% solid and is sheared, the coalescence of the material will break up, its viscosity will fall, and it will flow like a liquid, but if it is allowed to stand for a certain time, globular coalescence will increase the viscosity of the material, which leads to it being able to support its own weight and be handled in the same way as if it was solid [5, 6]. As described above, a globular microstructure with an appropriate amount of liquid fraction is required in order for SSM forming to be successful. When shear forces are applied, the near-globular particles move easily past one another, causing a decrease in viscosity and making the material behave like a liquid. In contrast, when shear forces are applied on dendrite microstructures, typical of conventional castings, the liquid is trapped between dendrite arms and prevents them from moving freely, thus increasing the viscosity of the material [7, 8].

Semisolid metal processing technologies can be grouped into two main categories based on the status of the starting material, liquid routes and solid routes. In recent years, a number of methods have been developed to make fine globular microstructures for feedstock production. Substantial efforts in research of feedstock production have been carried out over the years, and the intention of this paper is to review the various processes, highlight any differences between them, and present a review of the potential mechanisms that lead to microstructural alterations during the preparation of SSM slurries.

## 2. The Mechanism of Dendrite Fragmentation

**Figure 2:**
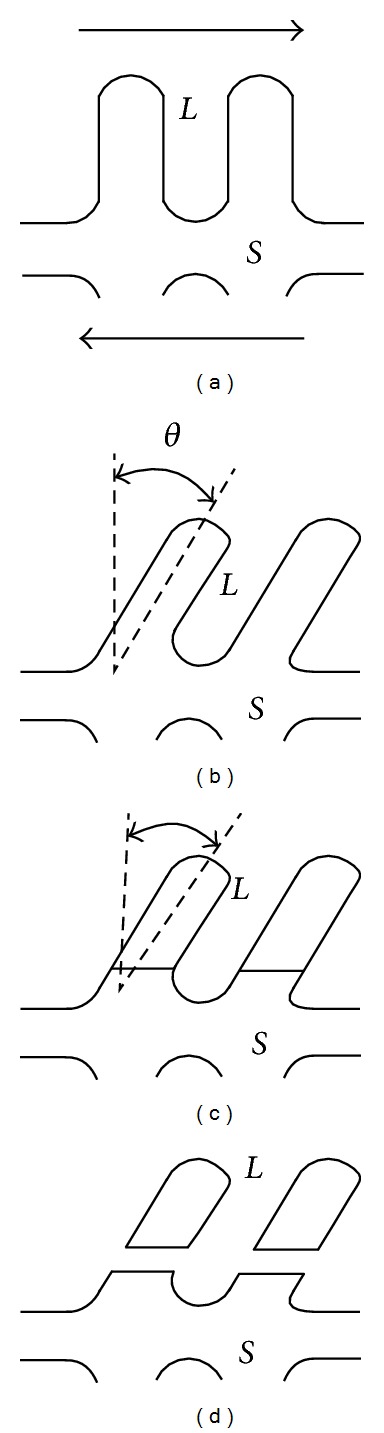
Schematic illustration of the steps of the mechanism of dendrite fragmentation: (a) undeformed dendrite; (b) after bending; (c) formation of high-angle boundary; (d) fragmentation through wetting of grain boundary by liquid metal [11].

Over the last 40 years, a number of mechanisms have been proposed by different researchers in order to account for the conversion mechanisms from dendritic to globular morphology. These mechanisms include dendrite arm fragmentation, dendrite arm root remelting, and growth control mechanisms. However, the identification of an accurate mechanism for the transformation of dendrites to a spherical morphology is still unclear [9]. Vogel et al. [10] suggest that dendrite arms have plasticity, which enables them to bend under shearing forces, thus introducing large misorientations inside the dendrite arms to form dislocations; at the melting temperature, rearrangements of dislocations occur to form grain boundaries. If the misorientations between grain boundaries are more than 20°, the energy of the grain boundaries becomes more than twice the solid/liquid interfacial energy, which leads to wetting of the grain boundaries by liquid metal and finally separation of the dendrite arms [11], schematically illustrated in Figure 2.

**Figure 3:**
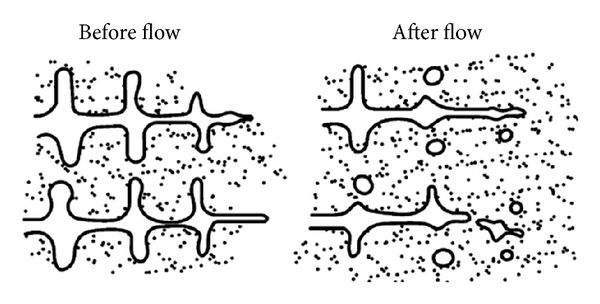
Schematic diagram of dendrite multiplication theory [15].

Hellawell [12] proposed that the solute enrichment and thermosolutal convection produced during solidification have a direct effect on secondary dendrite arms at their roots, which detaches them by melting off rather than breaking off [13, 14], thus yielding grain multiplication, schematically illustrated in Figure 3 [15]. The laminar flow adjusts the growth morphology from one that is regularly dendritic to one that is rosette in form, at the same time at which the turbulent flow adjusts the rosette growth morphology to one that is globular during solidification under forced convection [16].

## 3. Technologies for Production of Nondendritic Feedstock 

As discussed above, production of feedstock with thixotropic properties is a key step for successful SSM processing. In addition we have seen that SSM technologies are divided into two main categories based on the starting material status: (1) either from a liquid alloy through controlled solidification (by activating crystal multiplication of a growing solid or by increasing the nucleation rate) under specific conditions, or (2) from the solid state through heavy plastic deformation and recrystallization [17]. There are many routes for feedstock production which have been developed over the last 40 years, and each one is based on the activation of established metallurgical phenomena that determine the formation of the final structure [18]. The most effective methods, as well as those most commonly used in commercial practice, are described briefly below.

### 3.1. Mechanical Stirring

**Figure 4:**
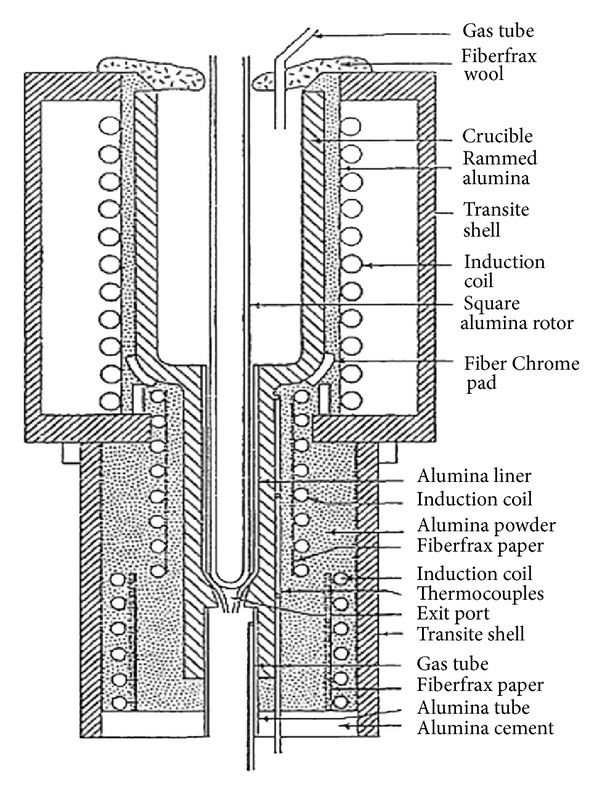
Schematic diagram of high-temperature continuous mechanical rheocaster [19].

**Figure 5:**
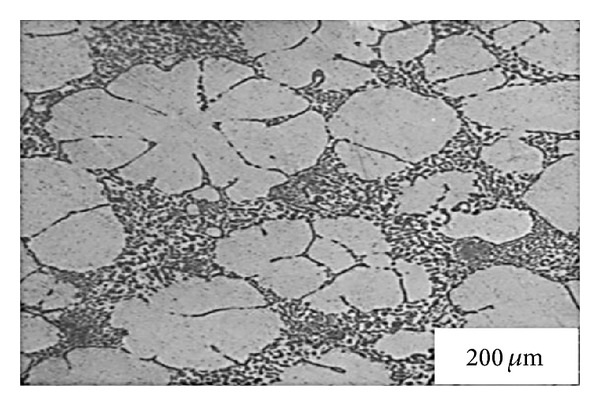
Microstructure of Sn-15% Pb alloy billets obtained by mechanical stirring [19].

This technique, originating at MIT, involves vigorous stirring of the melt contained in a holding vessel, using an auger, paddle, or impeller agitator during solidification in order to obtain a nondendritic microstructure, as shown in Figure 4. The feedstock from this method can be used directly in the semi-solid slurry state for near-net shaping of parts through the rheocasting route or through the thixoforming route that involves complete solidification of the feedstock which is reheated at a later time to the semi-solid state and shaped by either injecting into a die or by forging into the required shape, called thixocasting and thixoforging, respectively. The vigorous agitation of the superheated molten metal during solidification is responsible for the deformation and melting of dendrite arms that go to form equiaxed grains in the liquid matrix. These grains will remelt due to the surrounding bulk liquid that still contains small areas of superheated liquid within it. Only a small amount of particles continue to exist in the molten metal. These superior particles are developed after taking the temperature out of the molten metal for a predefined period of time to solidify them into fine and non-dendritic microstructures. The use of high solidification rates with high shearing rates produces fine and well-rounded particles in the liquid matrix. However, this route is not desirable for all commercial applications because of the following disadvantages: the forming of rosettes and solid particles, risk of gas interruption, erosion of the stirrer especially with high-melting-point alloys, contamination of the slurry by chemical reactions and oxidation through stirring, and difficulties associated with controlling the process on an industrial scale [1, 8].  Figure 5 shows an example of the microstructure of Sn-15% Pb alloy billets made by the mechanical stirring method.

### 3.2. Magnetohydrodynamic Stirring

**Figure 6:**
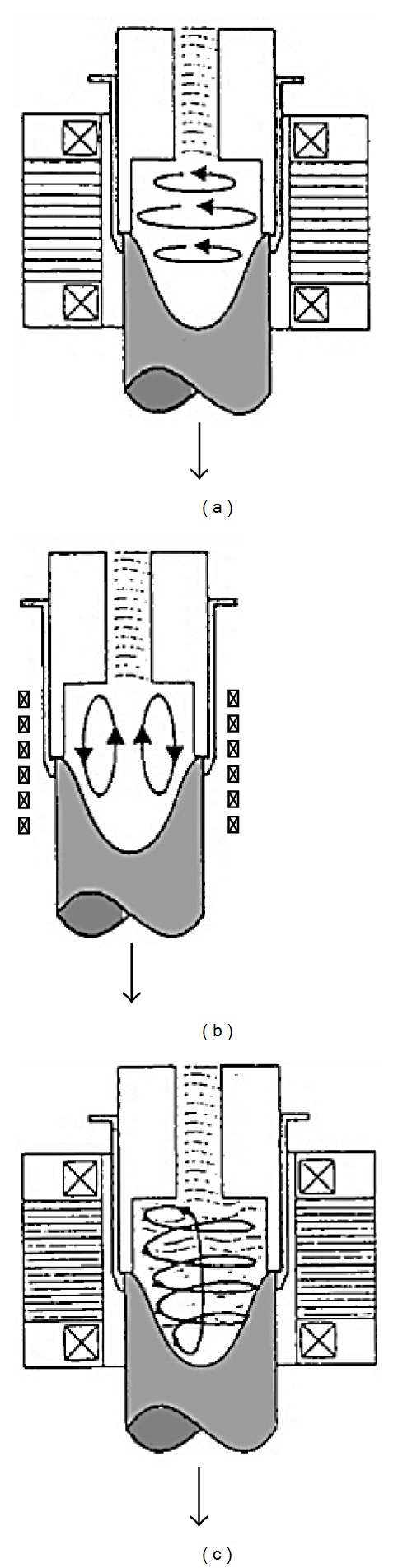
Schematic diagram of different flow modes system: (a) horizontal agitation; (b) vertical agitation; (c) helical agitation [23].

**Figure 7:**
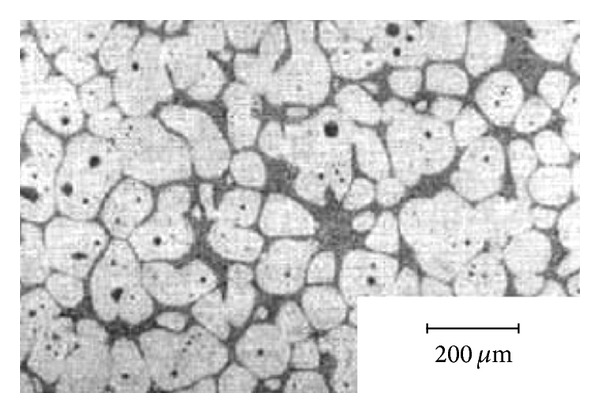
Microstructure of AlSi7Mg0.6 (A357) alloy billets obtained by MHD stirring [24].

In an attempt to get over the problems encountered with direct mechanical stirring, International Telephone and Telegraph (ITT) in the USA developed the magnetohydrodynamic (MHD) stirring process which can produce non-dendritic alloys through rotating electromagnetic fields that provide local shearing in order to break up the dendrites within a continuous casting mold. With this method, gas entrapment in the slurry is minimised, as is incorporation of any other extraneous matter, reducing contamination to a minimum. Furthermore, the slurry is filtered and degassed, generating large quantities of fine-grain feedstock material, typically 30 *μ*m, at fast production speeds, and of consistent repeatable quality, so important in commercial applications. For these reasons, the MHD stirring process quickly became the most widely used feedstock production method for thixoforming [20]. When electromagnetic stirring is applied to a melt near the freezing point at the surface of the chilled mold, a strong fluid flow in the semi-solid mushy zone creates the necessary shear stresses which are responsible for the deformation and melting of dendrite arms to form equiaxed grains in the liquid matrix. These grains will remelt due to the surrounding bulk liquid that still contains small areas of superheated liquid within it. As in mechanical stirring, only a small amount of the particles continue to exist in the melt. These particles are developed and solidified into fine, non-dendritic microstructures by removing heat from the melt for a predefined time [21, 22]. In this technology there are three modes that can be used to obtain rotational flow to achieve electromagnetic stirring: horizontal agitation, vertical agitation, and helical agitation, as shown in Figure 6. In the vertical agitation mode, the dendrites near the solidification area undergo convection transfer to the hotter zone to remelt and the globularization mechanism is controlled by thermal processing rather than mechanical shearing. In the horizontal agitation mode, the motion of the solid particles is held in an almost isothermal plane and the globularization mechanism is controlled by mechanical shearing. The helical mode is a combination of the horizontal and vertical modes. However, although this method is an improvement on mechanical stirring, there are still some problems, such as solid particles forming rosettes that are not completely round and the nonuniformity of the microstructure in the cross-section of the casting billet leading to increased reheating times. The production cost increases as a result of these problems, and the extra steps involved in feedstock production, as well as in actual processing of parts because of the reheating of feedstock necessary prior to forming into parts, and the difficulties of recycling the non-dendritic gates, off-cuts, and other scrap in situ, made the MHD route an expensive one. Nevertheless, MHD stirring has been the most effective and most commonly applied method for 20 years [23, 24].  Figure 7 shows an example of the microstructure of AlSi7Mg0.6 (A357) alloy billets obtained by MHD stirring.

### 3.3. Spray Casting

**Figure 8:**
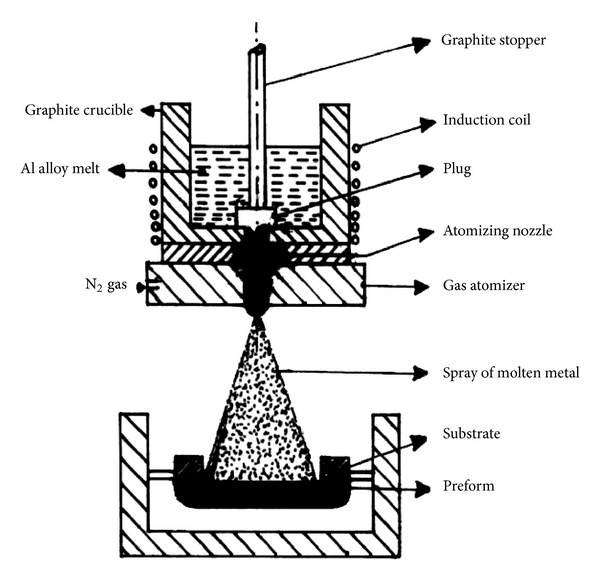
Schematic illustration of the experimental setup of spray forming [29].

**Figure 9:**
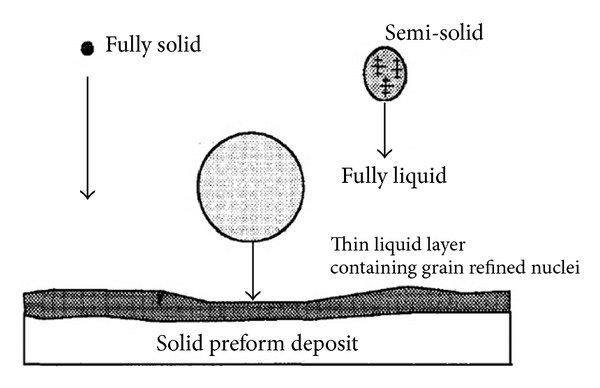
Schematic illustration of formation and structure of the spray process [25].

**Figure 10:**
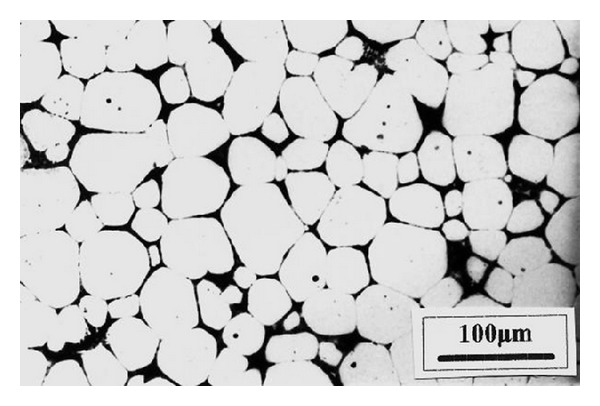
Microstructure of M2 tool steel obtained by spray forming [25].

Spray casting or spray forming is a nonagitation process for feedstock production that can be used to produce alloys which cannot be produced by any other route with a grain size of less than 20 *μ*m. Spray casting involves a gas jet that atomizes liquid metal into a stream of tiny droplets. These droplets can be produced in different sizes and are delivered onto a cooled target at high-flight velocities [25], as schematically illustrated in Figure 8. A second stage of solidification starts during atomization. At the beginning of the process, small droplets solidify directly, whereas large droplets are still fully liquid, while intermediate droplets are deposited in a semi-solid state on the upper surface of the preform [26], schematically illustrated in Figure 9. The mechanism for the formation of the resultant microstructure that develops from the dendritic droplets is difficult and complicated. It is suggested that dendrite arm fragmentation occurs due to the high impact velocity on the top surface of the intermediate droplets during their flight which causes them to spheroidize and coarsen at the top of the preform and grow before final solidification [27, 28]. Generally, this route is considered expensive and is less frequently used, and the billet sizes are typically not less than 60 mm. However, it is suitable for the thixoforming of high melting point alloys such as steel and superalloys [29].  Figure 10 shows an example of a microstructure of M2 tool steel made by the spray casting method.

### 3.4. Chemical Grain Refining

**Figure 11:**
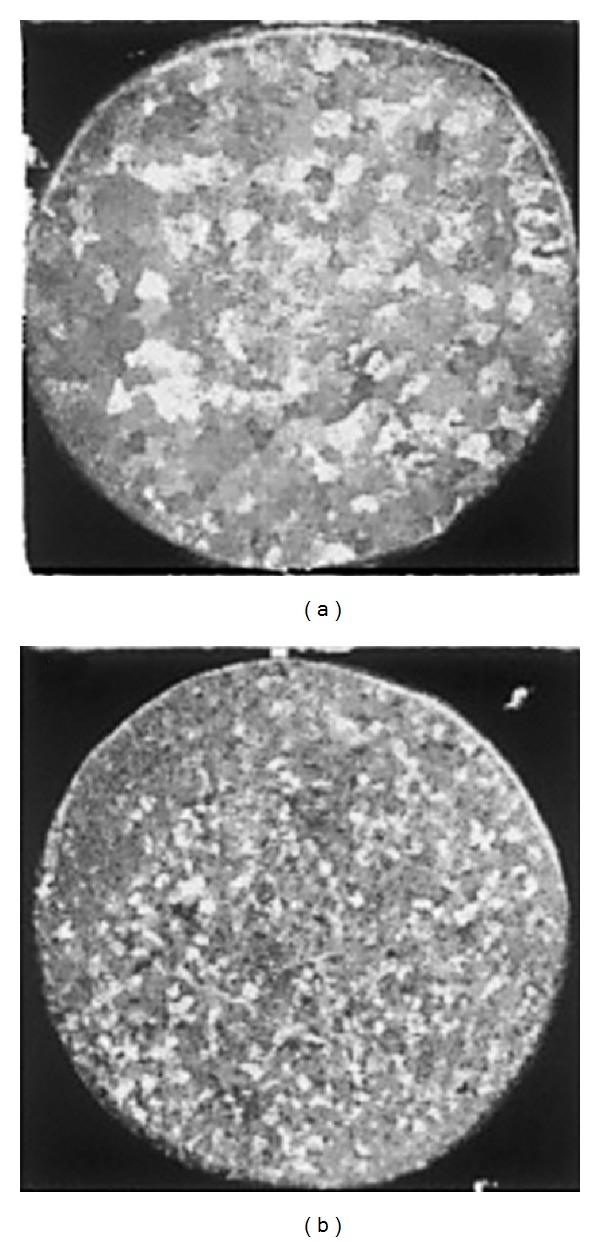
Microstructures of macroetched test samples of commercial purity Al: (a) unrefined; (b) refined by Al-5Ti-1B master alloy [39].

Chemical grain refining is a nonagitation process for feedstock production that involves an additional step in the process of continuous casting of alloys. This route is usually applied to specific types of alloy systems, such as Al-base alloys, by adding a Ti-B master alloy to the melt in order to support equiaxed grain growth rather than columnar growth prior to casting. The heterogeneous nucleation agent causes suppression of dendritic growth and produces equiaxed, fine grain size microstructures with better distribution of *α*-Al primary phases in the cast product or solidified ingot, as shown in Figure 11 [30]. This could be a perfect method for the inexpensive production of starting materials for semi-solid forming, creating particle sizes of around 100 *μ*m. However, this method cannot be used alone; it has to be combined with another route for feedstock production, such as liquid casting or MHD stirring [31]. The disadvantages of this method are that nucleation agents can work only on certain alloy systems and that an irregular spheroidal grain size with a high percentage of liquid fraction can occur, which negatively affects the quality of the process [2].

### 3.5. Liquidus Casting

**Figure 12:**
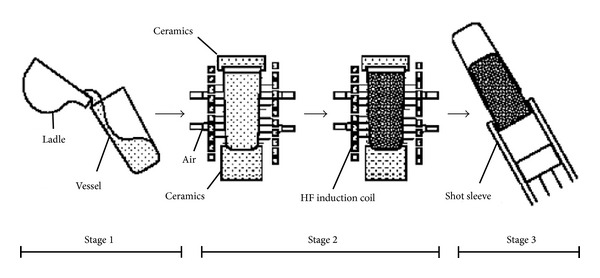
Schematic illustration of the stages of New Rheocasting (NRC) [40].

**Figure 13:**
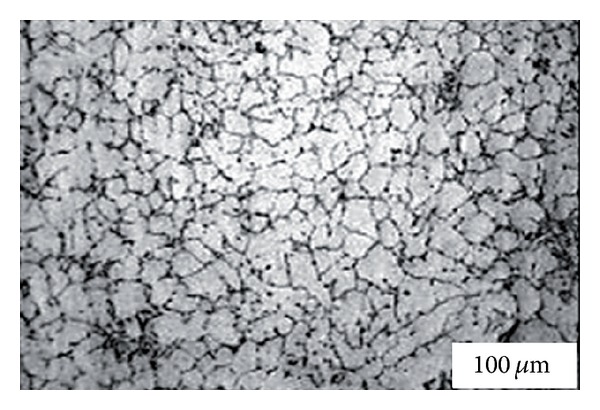
Microstructure of A356 alloy obtained by NRC method [41].

Liquidus casting, also known as a New Rheocasting (NRC) or low superheat casting, was developed as a low-cost alternative nonagitation technique for thixotropic feedstock production. New Rheocasting starts with the application of a low level of superheat to the melt at a uniform temperature near to or just above the liquidus temperature. Next, the molten metal is poured into a holding mold and kept there for a predefined time to be conditioned into slurries of fine, non-dendritic microstructures that can be charged into the inclined sleeve of a vertical squeeze casting machine, as shown in Figure 12 [32]. The mechanism of dendrite fragmentation in this technique can be explained as follows. The pouring of the superheated molten metal into the holding vessel is responsible for deformation and melting of the dendrite arms. The grains will remelt due to the surrounding bulk liquid that still contains small areas of superheated liquid within it. Nucleation takes place in the melt where only a small amount of particles continue to exist and these particles develop into fine, globular microstructures after the melt is undercooled for a predefined time [33], as shown in Figure 13. Two issues should be taken into account to ensure success when casting ingots using this technique. First, the melt superheat should not exceed 10°C, and second, the solidification speed of the molten metal has a direct effect on the shape of the initial crystal structures [34, 35].

### 3.6. Cooling Slope

**Figure 14:**
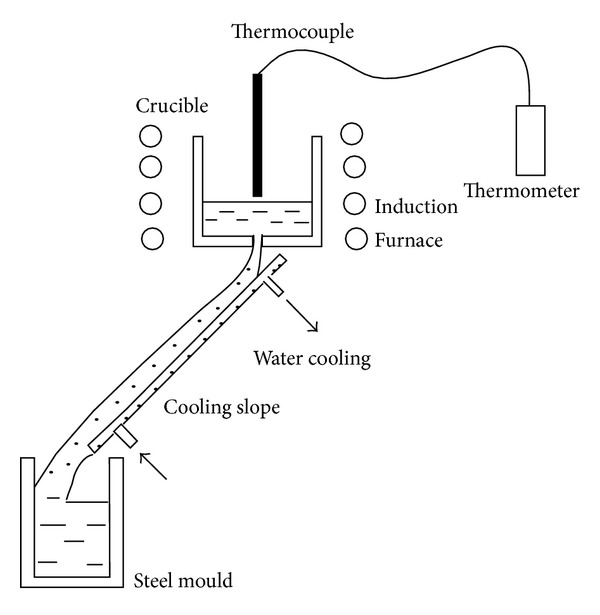
Schematic illustration of the stages of the cooling slope technique [35].

**Figure 15:**
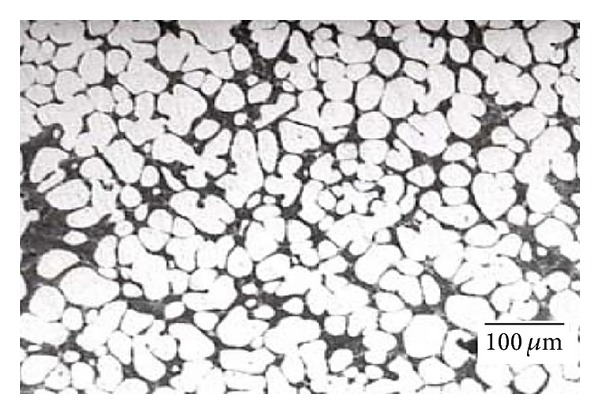
Microstructure of A356 aluminum at 620°C obtained by the cooling slope technique [35].

The cooling slope technique is the simplest nonagitation process that can be utilized to produce feedstock with a near-globular solid fraction in a liquid matrix. It is a continuous casting process that works by applying a low superheat to the metal at a uniform temperature near to or just above the liquidus temperature. Flow is achieved by pouring the molten metal into cold slope to gather and collect in a mold, or it can be used directly in combination with a shaping process such as rolling [36, 37]. The mechanism of dendrite fragmentation for this technique is based on crystal separation theory according to Motegi et al. [38]. The nucleation of granular crystals occurs at the contact surface of the cooling plate, and due to fluid movement, which is accelerated by gravity, the nucleation on the slope wall removes the molten metal into a heating mold, thus ensuring that the spheroid size is fine [38]. The process is shown in Figure 14, and the microstructure of A356 aluminum poured at 620°C and produced by the cooling slope method is shown in Figure 15. This type of process is not desirable for all applications for the following reasons: gas interruption, contamination of the slurry by the chemical reactions and oxidation, and difficulty of controlling the process. The principle variables that have a direct effect on the microstructure are the length and angle of the cooling slope, the molten metal superheating, and the material of the die [38].

### 3.7. New MIT

**Figure 16:**
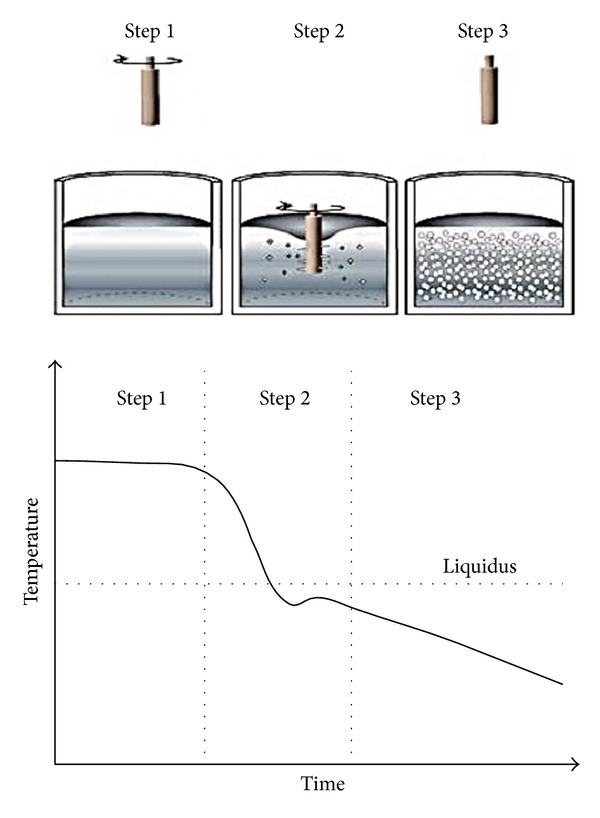
Illustration of the steps of the New MIT process [40].

**Figure 17:**
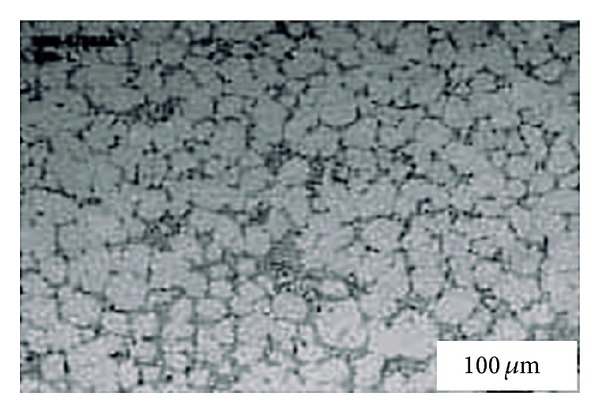
Microstructure of 356 alloy obtained by the New MIT process using air cooling as the stirring method [42].

New MIT is an agitation process which is a hybrid of stirring and near-liquid casting. This method was developed at MIT in 2000 and is known as New MIT or the Semisolid Rheocasting (SSR) process. The preparation of the slurry via this route can be broken down into three steps. The first step starts with holding molten metal slightly above its liquidus temperature to reach a homogenous temperature gradient. The second step is to insert a cool rod (normally made of graphite) into the melt to stir and rapidly cool the metal for a period until it reaches a temperature that is slightly below its liquidus temperature (i.e., to initiate solidification). The last step is to withdraw the stirrer and insert the metal directly into the casting device or cool it slowly to reach the desired solid fraction. The process, which is illustrated in Figure 16, produces fine, globular microstructures [31, 40]. The mechanism of dendrite fragmentation for this technique can be explained as follows. The cold graphite stirrer is implanted into the liquid metal, and the consequent low superheat temperature causes numerous fine dendritic grains to nucleate on the stirrer's surface. By stirring the melt, these particles are quickly removed and dispersed throughout the melt as very fine grains. These grains then remelt due to the surrounding bulk liquid that still has small areas of superheated liquid within it. Finer particles are then developed after cooling of the melt for a predefined time resulting into nondendritic microstructures [38]. A schematic view of the microstructure of 356 alloy obtained by the New MIT process, in this case using air cooling as the stirring method, is shown in Figure 17.

### 3.8. Swirled Enthalpy Equilibration Device Process

**Figure 18:**
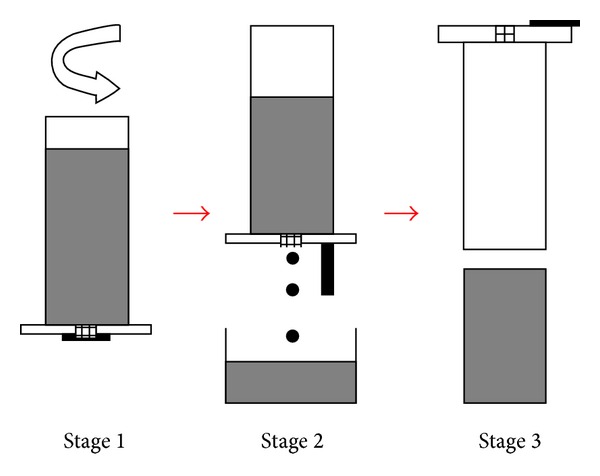
Schematic diagram of preparation procedure of slug in the SEED process [44].

**Figure 19:**
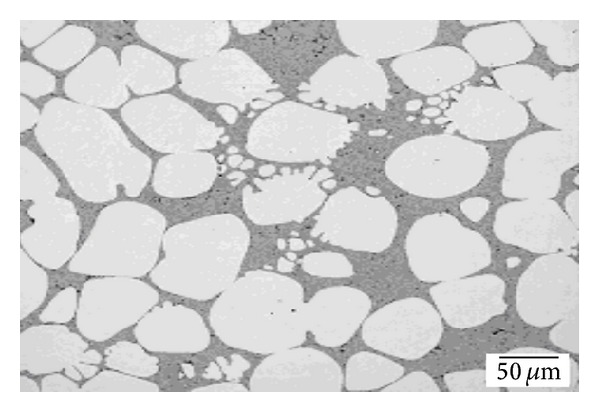
Microstructure of A356 aluminum obtained by the SEED method [44].

The Swirled Enthalpy Equilibration Device (SEED) process is a method for feedstock preparation for semi-solid forming processes patented by ALCAN International in 2002 [43]. It is based on the eccentric stirring of liquid metal in a metallic mold within a semi-solid mushy zone to initiate regular temperature distribution between the mold and bulk. The preparation of the slurry via this route can be broken down into three stages, as shown in Figure 18. The first stage starts with the application of a low superheat to the metal at a uniform temperature that is above the liquidus temperature followed by pouring the melt into a vessel (for heat extraction) whose thermal mass is sufficient to solidify the melt for a predefined time to achieve a specific solid fraction. During this stage the vessel and its contents are rotated at preset rpm according to the dimensions of the mold and the mass of the charge. The swirling action assists in ensuring that the solid phase is generated and several nucleation locations at the vessel surface are formed. In the second stage, the swirling action is stopped and a valve is opened at the vessel base to drain the remaining liquid in order to allow the primary particles to form until the material inside the container moves away from the container walls and becomes a self-supporting slug and recovers its semi-solid state, which can then be handled and formed under pressure into the preferred shape. The mechanism of dendrite fragmentation for this technique can be explained as follows. The swirling action assists in ensuring that several locations of nucleation at the vessel surface are formed, and this is followed by a slow cooling process to prevent the growth of dendritic arms. The microstructure of A356 aluminum obtained by the SEED method is shown in Figure 19. Two parameters should be taken into account to ensure success when casting ingots via this technique. First, the intensity of stirring, and second, the pouring temperature. These parameters directly affect the range of structures from dendritic to globular and the microstructural evolution [43, 44].

### 3.9. Direct Thermal Method

**Figure 20:**
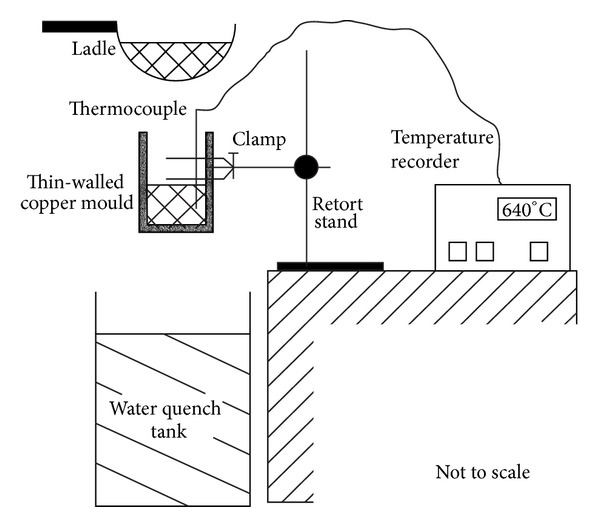
Schematic diagram of experimental procedure of the DTM [46].

**Figure 21:**
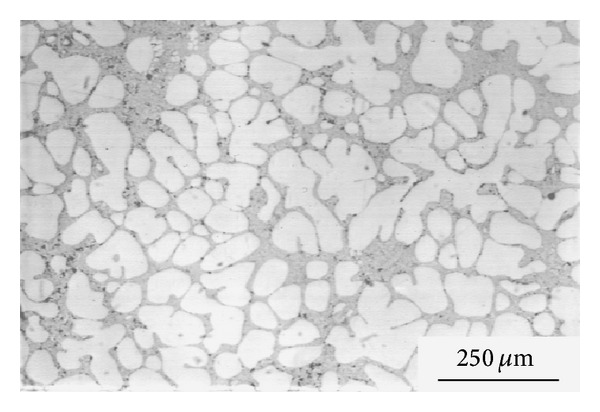
Microstructure of alloy A356 obtained by the DTM [46].

The Direct Thermal Method (DTM) of rheocasting was developed as an alternative technique for the production of thixotropic feedstock by University College Dublin in 2002 [45]. This process involves pouring low superheat liquid alloys into a thin-walled cylindrical mold with high conductivity and low thermal mass. At the first contact of the molten metal with the mold wall there is quick heat absorption from the melt to provide multiple nucleation. As a result of these conditions, the molten alloy cools very slowly at a very low rate by losing heat to the atmosphere to reach an equilibrium temperature below the liquidus (solidification range) of the alloy. The heat match between the mold and the alloy results in a pseudoisothermal holding state causing low heat convection transfer with a small thermal gradient [46]. Based on the preferred microstructure and the predefined temperature, the mold with its contents is quenched quickly in water. Basically, the mechanism of dendrite fragmentation for this technique is one that provides several locations of nucleation at the beginning of the process, followed by slow cooling to prevent the growth of dendritic arms without utilizing any special heating equipment or specific insulation [45], as shown in Figure 20. The microstructure of A356 aluminum obtained by the DTM is shown in Figure 21. This method is considered to be the cheapest and is ideal for laboratory environments that require limited sizes of billet [45].

### 3.10. Gas-Induced Semisolid Process

**Figure 22:**
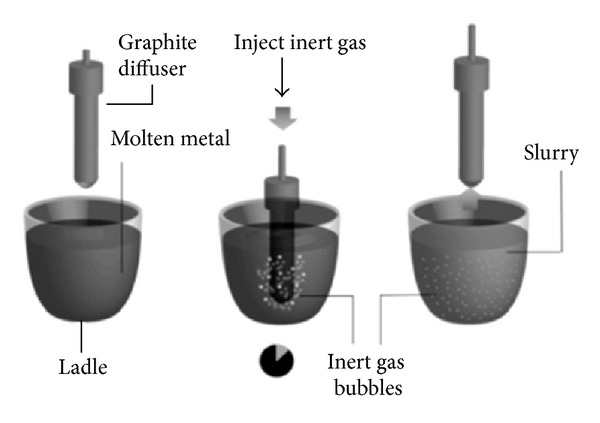
Schematic diagram of the steps of the GISS method [50].

**Figure 23:**
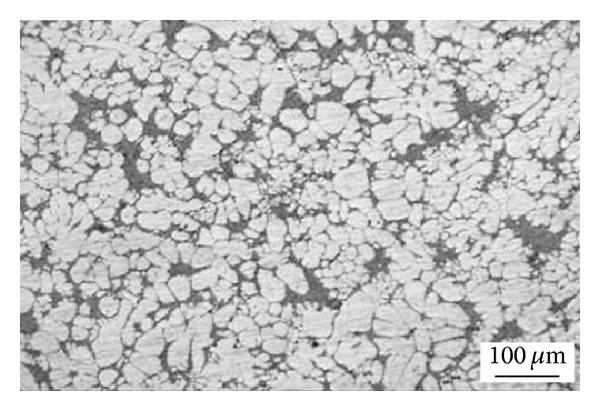
Microstructure of 356 aluminum alloy obtained by the GISS method [50].

A new idea for microstructure refinement to obtain a non-dendritic appearance exploits the theory of quick heat extraction and vigorous local extraction using the insertion of fine gas bubbles during solidification to stir up the melt as it cools down to the semi-solid range. The fine gas bubbles can be introduced in different configurations into molten metal in the gas-induced semi-solid (GISS) process [47–49]. The preparation of the slurry can be broken down into three steps in this method, as shown in Figure 22. The first step of the GISS method starts with the application of a low superheat at a uniform temperature near or just above the liquidus temperature of the metal. A porous graphite diffuser is then injected into the melt introducing inert gas bubbles. Finally, the diffuser is withdrawn and the semi-solid metal slurry is inserted directly into the casting device or cooled slowly until the preferred microstructure and solid fraction have been achieved, as shown in Figure 23. The mechanism of dendrite fragmentation for this technique can be explained as follows: the cold graphite diffuser is immersed in the melt, and the low superheat temperature causes numerous fine dendritic grains to nucleate and grow on the diffuser's surface. The flow of gas bubbles enables these particles to be speedily extracted into the melt. These fine grains remelt due to the surrounding bulk liquid which still contains small areas of superheated liquid within it. Only a small amount of particles continue to exist in the molten metal. These particles further develop by removing heat out of the melt for a predefined time to obtain fine, non-dendritic microstructures [50]. The GISS route is suitable for processing different types of alloys such as zinc alloys, cast aluminum alloys, wrought aluminum alloys, and die casting aluminum alloys [50].

### 3.11. Ultrasonic Vibrations

**Figure 24:**
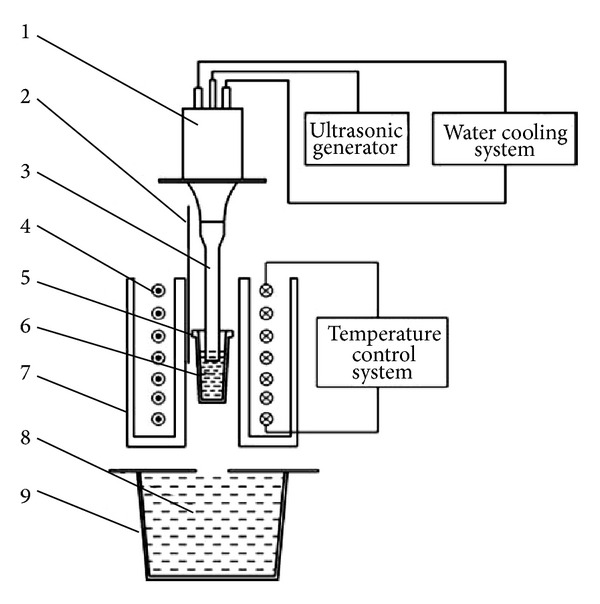
Schematic diagrams of the ultrasonic vibration device: (1) ultrasonic transducer; (2) thermocouple; (3) ultrasonic radiator; (4) resistance heater; (5) iron crucible; (6) Mg-9.0Al%; (7) ceramic tube; (8) water; (9) tank [52].

**Figure 25:**
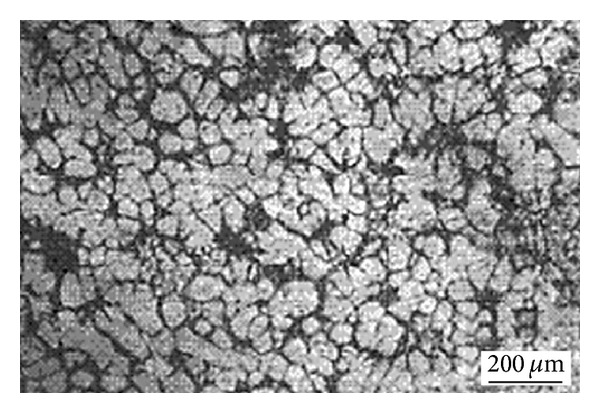
Microstructure of 390 aluminum alloy obtained by ultrasonic vibration [53].

Ultrasonic vibrations are an alternative method for the preparation of feedstock for semi-solid forming processes that was patented in the mid-1970s. It is based on applying high-power ultrasonic vibrations (high frequency mechanical waves) into a cooling melt to increase the number of solidification nuclei. The application of pulsed ultrasonic vibrations to the melt during solidification encourages cavitation that creates huge instantaneous fluctuations in the pressure and temperature of the melt to produce fine, globular microstructures [51], as shown in Figure 24. The microstructure of A390 aluminum formed using this method is shown in Figure 25. The mechanism of dendrite fragmentation for this technique is represented by two basic physical phenomena: cavitation and acoustic streaming. Cavitation includes the formation, growth, pulsating, and collapsing of tiny bubbles in the melt. The compression rate of these unsteady states can be so high that their collapse produces waves of hydraulic shock which in turn break up the primary particles and produce artificial sources of nuclei. The propagation of high-intensity ultrasonic waves also involves the initiation of steady-state acoustic streaming in the melt. The total effect of these different types of streams is to vigorously mix and thus homogenize the melt [51]. When ultrasonic vibrations are applied during solidification the microstructure can be summarized as follows: decrease of mean grain size, segregation control, and variation of phase distribution with better material homogeneity so that products show superior mechanical performance [52, 53].

### 3.12. Shearing-Cooling Roll

**Figure 26:**
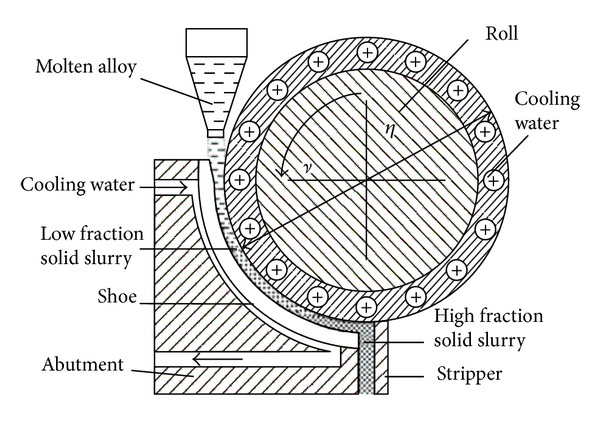
Schematic diagram of SCR process [55].

**Figure 27:**
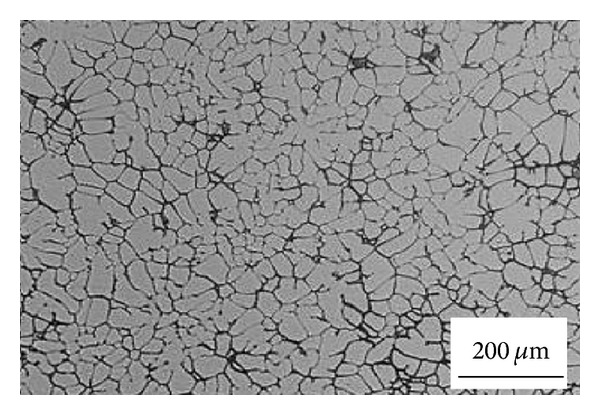
Microstructure of A2017 aluminum alloy obtained by SCR process [55].

The Shearing-Cooling Roll (SCR) process is an effective, liquid-state process with commercial potential that has been developed as an alternative technique for the production of thixotropic feedstock. The working principle of preparing semi-solid alloy by means of the SCR process is shown in Figure 26. Briefly, this process is based on applying superheat to the alloy melt at a uniform temperature near or just above the liquidus. Flow is created by pouring the molten metal into the inlet of a rotating roll and a stationary cooling shoe cavity, creating frictional forces as a result of the rotating roll. At the roll-shoe cavity, the molten alloy is cooled steadily by the shoe and the roll, and through the shear forces produced, generates globular, semi-solid slurries [54]. The formation and evolution of non-dendritic microstructures in the SCR process can be explained as follows. When the molten alloy is cooled at the contact surface of the roll-shoe, there is nucleation of granular dendritic crystals. These, under the shearing force and the stirring of the roll during solidification of the liquid alloy with high solid fraction, are crushed and disperse into the melt to form equiaxed or spheroidal grains. In the SCR process, the use of high solidification rates with high shearing rates can produce fine and well-rounded particles in the liquid matrix [55]. The microstructure of A2017 aluminum produced by SCR method is shown in Figure 27.

### 3.13. Stress-Induced and Melt-Activated Process

**Figure 28:**
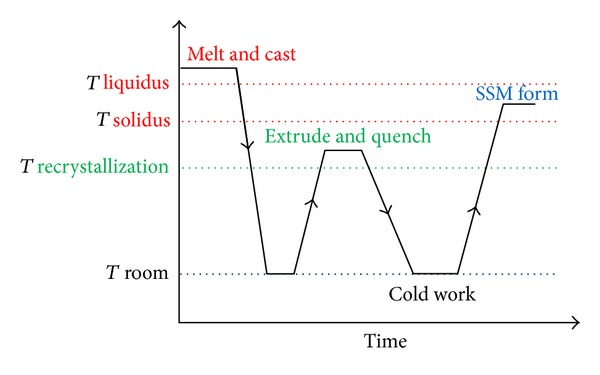
Schematic illustration of the stages of the SIMA process [56].

**Figure 29:**
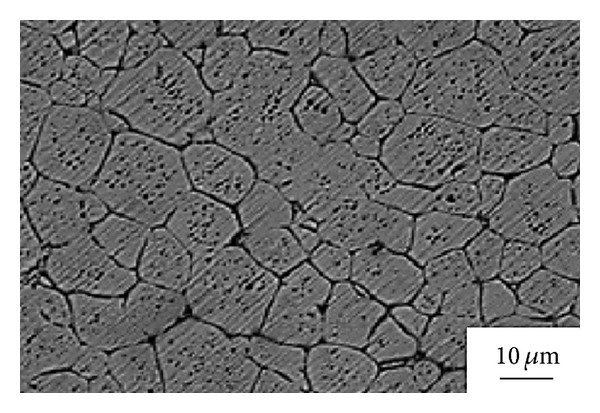
Microstructure of deformed Al 7075 alloy obtained by the SIMA process in the case of being isothermally held for 5 min at 620°C [58].

The Stress-Induced and Melt-Activated (SIMA) process is one of the most effective and commercially available solid state processes. Briefly, the procedure of this technique relies on reheating deformed material into the semi-solid state. The initial deformation occurs above the recrystallization temperature (hot working). Then there is cold working at room temperature, which is a critical step in storing the energy flow as result of partial remelting whereby the material reaches a semi-solid state to obtain uniform, non-dendritic, spherical solid particles within a liquid matrix, as shown in Figure 28. The evolution of non-dendritic microstructures during this process occurs as a result of the partial re-melting that is applied to the deformed material to recrystallize and obtain fine, non-dendritic microstructures through a process of liquid penetration of high-angle grain boundaries. When recrystallization is achieved and the liquid metal with high-energy infiltration flow through the high-angle boundaries of grains in order to evolve globular solid particles in a liquid matrix. The size of the globular solid particles has a direct relation with the rate of heating and the degree of cold work. The resultant particles can be as small as 30 *μ*m. This route seems to be a good method and a potential competitor to the MHD process [56, 57]. The microstructure of A7075 aluminum produced by using the SIMA process is shown in Figure 29.

### 3.14. Recrystallization and Partial Melting

**Figure 30:**
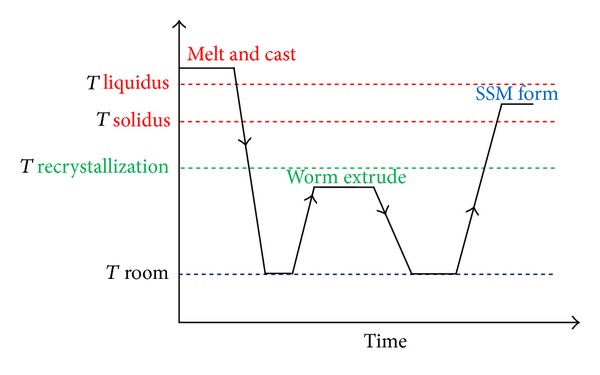
Process stages of the RAP method [60].

**Figure 31:**
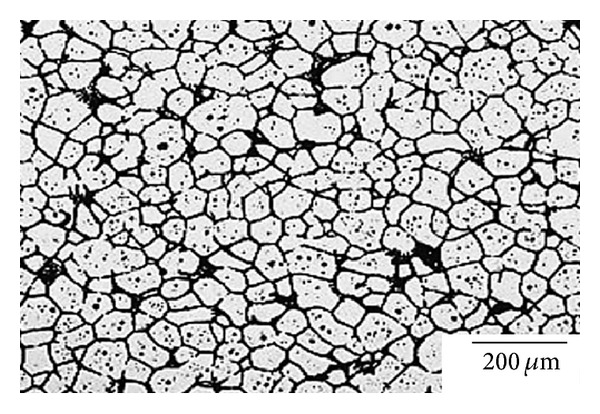
Microstructure of 7075 aluminium alloy obtained by the RAP method [61].

The Recrystallization and Partial Melting (RAP) method is similar to (SIMA), with the essential difference that initial deformation occurs below the recrystallization temperature, that is, during cold working. Fragmentation occurs as a result of high-energy liquid metal flow through high-angle grain boundaries to obtain fine, non-dendritic microstructures. This technique was established by Kirkwood and coworkers when they changed the cold work to warm work at a temperature below the recrystallization temperature to ensure maximum strain hardening [59], as schematically illustrated in Figure 30. For effective application, the following processing parameters need to be understood: duration of heating, reheating temperature, amount of plastic deformation, and how these can affect the microstructure in the semi-solid state [60]. The microstructure of 7075 aluminium alloy produced by the RAP method is shown in Figure 31.

### 3.15. Direct Partial Remelting

**Figure 32:**
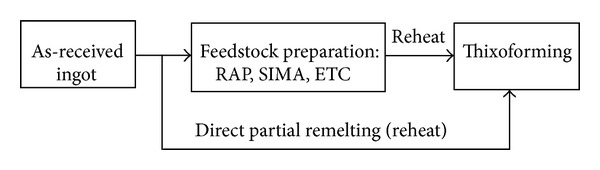
Schematic diagram of the different routes to obtain suitable thixformable microstructures.

**Figure 33:**
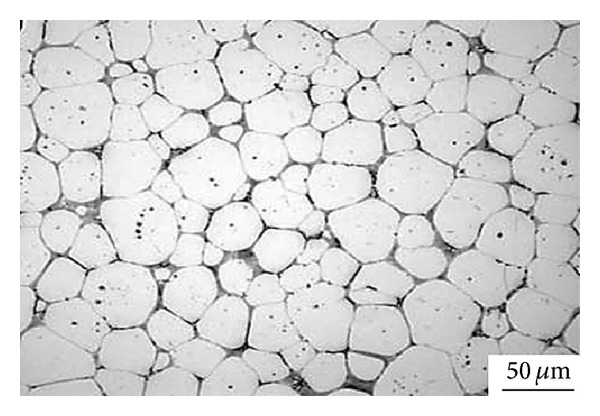
Microstructure of as-supplied M2 tool steel obtained by the DPRM [68].

The Direct Partial Remelting method (DPRM) is considered one of the most effective and commercially viable solid state processes (especially high-melting-point metals) to produce a non-dendritic microstructure in order to gain some insight into the microstructural development of the starting material when in the semi-solid state [62, 63]. The partial remelting experiments can be directly reheated to their semi-solid range from the as-received state without having to go through the conventional feedstock preparation routes, like SIMA, RAP, and various others, as summarised in Figure 32. This indicates a widening of the range of potential routes to thixoformable microstructures [64–67]. A schematic view of the microstructure of M2 tool steel produced by using the DPRM is shown in Figure 33.

### 3.16. Solid-Solution Treatment and Partial Remelting Method

**Figure 34:**
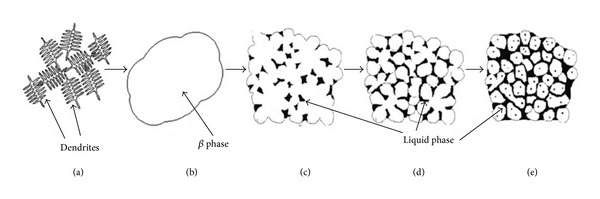
Schematic illustration of microstructural evolution from the as-cast sample: (a) the initial dendritic structure; (b) homogeneous *β* phase after solution treatment at 330°C for at least 3 h; (c)–(e) microstructures when (b) was partially remelted at 438°C for 15, 30, and 60 min, respectively [69].

**Figure 35:**
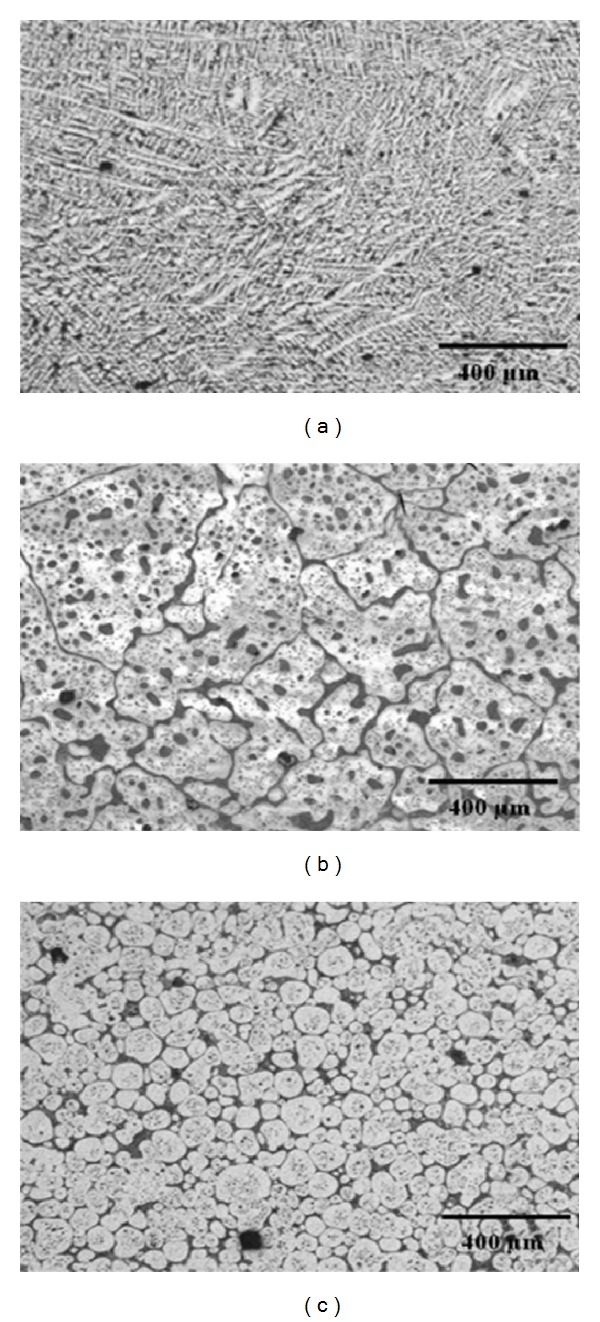
Microstructures of Zn-22Al (a) as-cast, (b) after DPRM at 438°C for 60 min in an as-cast sample, and (c) after solution treatment for 3 hours then subjected to DPRM at 438°C for 60 min [69].

The solid-solution treatment and partial re-melting method is similar to DPRM with the essential difference that the direct partial remelting into its semisolid range occurs after applying isothermal heat treatments (solid-solution treatment) not from as-received condition. In other words, a semisolid for some alloys with solid spherical particles could not be obtained if the traditional cast alloy was directly subjected to partial remelting. Whilst, when the alloy was solid-solution treated for sufficient holding time prior to partial remelting, small spherical structures were obtained. Subsequent solid-solution-treatment may result in the dissolution of the interdendritic eutectics, and the dendritic structures gradually transformed into uniform structures. The coarsened and merged dendrites were discrete as a result of the liquid phase penetration and melting of the residual eutectic next to the former grain boundaries. These nondendritic microstructures may be suitable for semisolid metal processing applications.  Figure 34 is a schematic illustration of the proposed sequence of structural evolution during the solution treatment of samples subjected to partial remelting. Arif et al. have reported some work on the semisolid microstructure evolution of Zn-22Al alloy [69]. This alloy was subjected to partial re-melting at 438°C for 60 min in an as-cast sample and in samples previously solution treated at 330°C for 3 hours as shown in Figure 35. The microstructure of the solid-solution-treated sample transformed into a small globular structure; the best shape factor of 0.9, corresponding to a particle size of 40 *μ*m, was achieved. 

## 4. Discussion

It has been shown that a number of mechanisms have been proposed in order to identify the processes and means by which dendrites can be converted into globular morphologies. Semi-solid metal processing technologies can be categorized into two major groups based on the starting material status: (1) liquid routes, which in turn can be divided into agitation and nonagitation routes and (2) solid-state routes. In the case of the former, it can be seen that some of these routes are used to produce alloy feedstock because there are no other means to achieve the non-dendritic fine grain size necessary, especially with respect to the thixoforming of high-melting-point alloys such as steel and superalloys (i.e., spray casting).

The mechanical stirring processing route was developed as a “batch” process rather than continuous production route, and although useful for small-scale laboratory experimentation it does not provide a sound basis for large-scale industrial production.

In order to enhance the potential of processing alloys in semi-solid state and also to overcome the problems associated with direct mechanical stirring, a magnetohydrodynamic (MHD) caster was developed. This route is suitable for continuous casting of large quantities of good repeatable quality feedstock, appropriate to commercial industrial applications but carries an additional cost penalty and any scrap is not recyclable in situ. A major advantage of the MHD process comes from the fact that the slurry is in the semi-solid state, rather than the semiliquid state. This allows better control of any surface oxides and results in products with better mechanical properties than the NRC, SSR, or the GISS liquid routes. As a result MHD is the preferred route for critical components where mechanical properties are at a premium.

Semisolid Rheocasting (SSR) is a hybrid of stirring and near liquidus casting. In the SSR process, molten metal is rapidly cooled via spinning cold finger which acts as a stirrer. This process has a good potential to be used in industrial applications, has lower costs, and is fully recyclable, and although it will give better properties than conventional casting routes, it will not reach the properties of the MHD route, while SEED process was introduced by Alcan and this process helps to overcome problems with high feedstock costs.

The UBE New Rheocasting process is based on the principle of feedstock production through manipulation of solidification conditions. The process was introduced to allow recycling of non-dendritic scrap material in situ, and although it does deliver on this, it is cumbersome and costly and its commercialisation was therefore short lived.

Cooling slope casting is a method to produce raw material required for thixoforming. The technique is simple but it can be hampered by the introduction of defects and inclusions that render the process unsuitable for critical applications. As other liquid route processes it will offer improvements on conventional casting techniques and is recyclable.

In contrast, solid state techniques have a number of advantages over liquid agitation methods because they are easier, require less equipment than the stirring or the MHD methods, and of course any deformed material off the shelf is potentially an appropriate SSM feedstock. The prominent solid-state routes are Direct Partial Remelting method (DPRM), Strain Induced Melt Activated (SIMA), and Recrystallization and Partial Melting (RAP) have been successfully used in commercial applications.

The SIMA process produced microstructures with near-spherical grains, most suitable for semisolid processing. This process produces high-quality feedstock for thixoforming and has the potential for wrought alloys and high-melting-temperature alloys such as steel and super alloys. The RAP is limited to small billet sizes due to the requirement of high and uniform deformation over the whole cross-sectional area. The advantages of these routes are that many alloys are usually supplied in the extruded state and the spheroids tend to be more rounded giving better flow than the rosette-type structures found in other feedstock routes, that is, such as the MHD route.

DPRM can be directly reheated to its semi-solid range from the as-received state without having to go through the conventional feedstock preparation routes. In addition, solid-state routes can provide more uniform and more rounded particles when compared with the particles that can be obtained by, for example, the MHD route.

Some alloys may not transform into globular structures when subjected to direct partial remelting from their as-cast state, as shown by Zn-22Al zinc alloy. However, when subjected to solution treatment (that resulted in some phase changes to the material), small near-spherical structures can be achieved. Overall, it might be true to state that the solid-state routes could be the most effective method for producing feedstock material for thixoforming high-melting-point alloys.

## 5. Conclusions

1. Semi-solid metal processing technologies can be categorized into two major groups based on the starting material status: (1) liquid routes, which in turn can be divided into agitation and nonagitation routes and (2) solid-state routes.
2. Mechanical stirring, magnetohydrodynamic stirring (MHD), Semisolid Rheocasting (SSR), and SEED (Swirled Enthalpy Equilibrium Device) are categorized as agitation routes, whereas UBE's New Rheocasting (NRC) and Cooling Slope casting as nonagitation routes. The main solid-state routes are Strain Induced Melt Activated (SIMA), Recrystallisation and Partial Melting (RAP), Direct Partial Remelting (DPRM). Other semisolid processing variants are thixoforming, thixoforging, thixocasting, and thixomolding.
3. Rheoroutes involve preparation of SSM slurry from the liquid phase and its transfer into a die or mold for near-net shaping. Thixoroutes involve feedstock material with near-globular microstructures which is injected into the mold after reheating and holding at an appropriate temperature between solidus and liquidus in order to obtain the necessary fraction liquid. Microstructures that are most suitable for thixoforming and will result in products of good mechanical properties are those that have fine homogenous grain size, high roundness shape factor, that is, approaching 1.
4. The main routes for slurry preparation have been discussed together with the mechanisms responsible for the formation of near-globular microstructures. This review comprehensively illustrates the scope of investigations in this field to date and concludes that some of the main issues and challenges to be considered when generating non-dendritic feedstock production methods are as follows
  - *Performance*. The most important issue in semi solid metal processing is the production of fine near-spheroidal microstructures. In this respect, MHD has been the most efficient method able to produce large quantities of consistently good quality globular microstructures appropriate for commercial applications as compared to other techniques.
  - *Simplicity*. Although simplicity of the technique should play an important role in the final choice, for example, Mechanical Stirring and Cooling Slope are simple techniques but their lack of complexity comes together with major limitations such as oxidation and gas porosity, thus making them less favourable for commercial applications.
  - *Cost*. Spray casting technique is an expensive route for non-dendritic feedstock production, but on high value, high quality critical applications such as aerospace this cost might be justifiable. Feedstock costs must always be considered as part and parcel of the whole process, including the recycling of scrap.
5. Over the years, great understanding of the grain morphology transformation process and the processing parameters required for successfully processing alloys and composites in the semi-solid state has been gained through the work of researchers across the world. Nevertheless, these interesting metal-processing routes, with the exceptions of two success stories, thixomolding users and V-forge Inc. in USA that uses MHD feedstock, have not yet fulfilled their potential in bridging the gap between casting and forging. This lucrative market is still up for grabs, and although thixoforming still has the potential to get there, it now faces new competition from additive manufacturing technologies such as Shape Metal Deposition or Metal Injection Molding.
